# Voices from the past: economic and political vulnerabilities in the making of next generation EU

**DOI:** 10.1057/s41295-022-00277-6

**Published:** 2022-03-07

**Authors:** Klaus Armingeon, Caroline de la Porte, Elke Heins, Stefano Sacchi

**Affiliations:** 1 University of Zurich, Affolternstrasse 56, 8050 Zürich, Switzerland; 2 Copenhagen Business School, Porcelænshaven 24, 2000 Frederiksberg, Denmark; 3 School of Social and Political Science, University of Edinburgh, 15A George Square, Edinburgh, EH8 9LD UK; 4 Department of Management and Production Engineering, Polytechnic University of Turin, Corso Duca degli Abruzzi 24, 10129 Turin, Italy

**Keywords:** European Union, COVID-19, NGEU, Germany, Italy, The Netherlands

## Abstract

In this article, we show that Next Generation EU (NGEU) is mainly a response to the economic and political imbalances left over from the Eurozone crisis. It is a pre-emptive intervention, especially targeted at structurally weak economies with rising Euroscepticism, to avoid costly ex-post bailouts as in the Great Recession. We demonstrate, using quantitative analysis, that pre-existing vulnerabilities, rather than the impact of the pandemic, drove the allocation of NGEU resources: per capita grants largely correspond to past economic vulnerabilities, as well as to political ones. Countries most vulnerable to another adjustment by austerity after the COVID-19 economic crisis receive most resources. Also, countries with strong anti-EU sentiments are entitled to larger NGEU grants per capita. In contrast, grants are not correlated with the severity of the health crisis. Then, we show the domestic relevance of economic and political vulnerabilities through qualitative case studies of national political debates and domestic positions on NGEU in Italy, Germany and the Netherlands. Despite its innovative traits, NGEU is a politically constrained solution to address the mess from the previous decade, and as such, it is a Janus solution: promising a fresh start, but haunted by the past.

## Introduction

The European Council decision in July 2020 to stimulate economic recovery from the COVID-19 pandemic with the 750-billion-euro Next Generation EU (NGEU) package could mark a critical juncture in the development of the European Union (EU) towards a stronger redistributive function (Jones 2021; Rhodes 2021; Schelkle forthcoming). For example, Buti and Papaconstantinou (2021) maintain that the European Council decisions “represent a clear break with precedent”, in terms of instruments, institutional mechanics and “the sheer magnitude of the underlying fiscal effort and liquidity provision”. Crises, such as a pandemic, have widely been identified as opportunities for learning (Kamkhaji and Radaelli 2017). With respect to NGEU, Ladi and Tsarouhas (2020) argue that policy learning from previous crises by national and supra-national actors led to profound changes, which modified the norms, policies and objectives of the EU. This is presented as a contrast to the crisis response in 2010, which has been characterised in the literature as a mere continuation, and even strengthening of the past framework governing the Eurozone, due to the political interests of member states (Dyson 2014; Lehner and Wasserfallen 2019).

There was indeed a swift reaction with a proactive fiscal programme and with some important taboos seeming to have been broken (including as regards common debts and bonds issued by the Commission, which had been abhorred during the Eurozone crisis by the so-called ‘creditor countries’). Under COVID-19, the Commission used open windows of opportunity to push forward programmes it had nurtured for some time, notably regarding its own resources and its ambitions for the green and digital transformations.^1^ In addition, the type of conditionality that underpins the receipt of funds from the Recovery and Resilience Facility (RRF), which constitutes the lion’s share of the NGEU package, is also seemingly new.^2^ During the Great Recession, conditionality was *austerity-oriented*, implicitly in Italy (Sacchi 2015), but usually explicitly and set out in covenants (in Cyprus, Greece, Ireland and Portugal). For the disbursement of loans, member states had to implement sweeping structural reforms and social spending cuts that were agreed under the terms of the Memoranda of Understanding signed between national governments and the Troika of the EU, European Central Bank (ECB) and International Monetary Fund (Armingeon and Baccaro 2012; de la Porte and Heins 2015; Dukelow 2015; Moury and Standring 2017; Theodoropoulou 2015). In contrast, RRF conditionality can be described as *expansionary-oriented*: to receive funding, member states must prepare national plans in line with a forward-looking EU agenda based on the European Green Deal, Europe’s new growth strategy (European Commission 2019). This includes the requirement to commit at least 37% of funds for green growth and 20% for digitalization programmes in member states’ national RRF plans. The remaining RRF funds have to be used for policies that support economic recovery and resilience, such as upskilling and innovation, consistent with Country Specific Recommendations (CSRs) proposed by the Commission and adopted by the Council as part of the European Semester process that governs EU socio-economic governance (European Commission 2020a; European Parliament and European Council 2021). Since NGEU is the EU’s response to help repair the economic and social damage caused by the pandemic crisis (European Commission 2020; European Council 2020), we would expect that the programme would reflect the severity of the pandemic, including its economic and social consequences. Surprisingly, there is a limited orientation to the actual effects of the health crisis, even if measured at the time NGEU was decided.

In this paper, we show that NGEU is mainly a response to the economic and political imbalances left over from the Eurozone crisis. This is evident from the allocation formula of the RRF: the grants have been based on economic criteria and population size, while the real immediate effects of the pandemic, such as regarding excess mortality rates, the number of pandemic-related bankruptcies or the increase in income inequality and poverty, never entered the allocation key agreed by the European Council when determining the size of grants (see European Parliament and European Council 2021; Darvas 2020). While the second tranche of NGEU (30% of grants) takes the fall of GDP since the onset of the crisis into account, in addition to population size and GDP per capita, the first tranche (70% of grants) is exclusively based on pre-crisis economic conditions, in particular a member state’s average unemployment rate over the period 2015–2019 relative to the EU average over this period. In response to the pandemic, the decision-makers’ major concern was to deal with the economic and political consequences of the mismanagement of the sovereign debt crisis, in order to avoid a break-up of the Eurozone and further economic fragmentation and political instability within the EU. Seen from this angle, we highlight path-dependence in the configuration of NGEU, mainly as its origins can be traced to the post-2008 financial crisis, rather than in the novelty or real impact of the pandemic crisis. In this regard, our analysis complements other analyses of NGEU that have considered the asymmetric nature of the architecture of Economic and Monetary Union (EMU) architecture (Howarth and Quaglia 2021), broadly resonating with a ‘failing-forward’ analytical framework (Jones et al. 2015). At the same time, we highlight how NGEU can be seen as a ‘deliberate attempt at changing the path of bailout funding’ (Schelkle forthcoming), addressing the interplay of the pandemic with the economic and political leftovers of the Great Recession, but this time via pre-emptive intervention, or ‘ex-ante’ rather than ex-post bailouts*,* the latter of which had a devastating impact on political support for the EU in some crisis-hit economies (Armingeon et al, 2016).

This paper is organised as follows: we start by showing that claims about the novelty of the NGEU are overstated. Thereafter, we specify our key inter-related concepts relating to economic and political vulnerabilities which are central for the survival of the eurozone and, by extension, the EU.^3^ Subsequently, we undertake our empirical analysis. In the first part, we examine economic and fiscal vulnerabilities of member states in the Eurozone crisis and the ongoing pandemic crisis, and the ensuing political vulnerabilities which have emerged. We find that pre-existing vulnerabilities, rather than the impact of the pandemic, drove the allocation of NGEU resources, with RRF (per capita) grants largely corresponding to past economic vulnerabilities, as well as to political ones. In the second part, we illustrate with three country case studies, representing distinct economic and political vulnerabilities, how NGEU responds to ‘voices from the past’. We show how this takes place in the context of ‘constraining dissensus’ (Hooghe and Marks 2009); however, political leaders still retain room for manoeuvre and ‘steering capacity’ (Ferrera et al. 2021). We thus conclude that NGEU was shaped by past vulnerabilities, rather than addressing the pandemic per se.

## Another reading of NGEU

There is no reason to belittle the achievements of heads of state and government and the European Commission in developing a response to the COVID-19 crisis (Schmidt 2020). The gross figures of the programme in addition to the multi-annual budget look impressive. However, any claim of a massive and historic policy change must be weighed against and possibly reconsidered in light of the following:

1. Claims of joint debt liability are exaggerated. The repayment of the common ‘Corona debt’ is a fixed expenditure within the EU budget. The member states are only liable via the EU's own resources, i.e. the contributions based on gross national income that each member state transfers to the EU budget. The measures are also explicitly temporary and limited in scope (European Council 2020). Hence, the taboo of common debts is only dented, but not broken. In addition, ‘on several occasions the Union (or the Community) borrowed on the capital market even before the current NGEU program’ (Tosato 2021, p. 2).
2. The overall size of the stimulus within NGEU is moderate. Under its terms, up to €750 billion (including €390 in loans and €360 in grants), as agreed in July 2020, would be distributed over three years, i.e. €250 billion per year. With a cumulative EU GDP of €13,965 billion, the average annual stimulus would represent around 1.8% of GDP^.^^4^ However, if the loans are not drawn down in full, the annual stimulus would be even lower (Jones 2021).^5^ In comparison, the US stimulus of 1.9 trillion US$ adopted in March 2021 is equivalent to 9% of US GDP (Stiglitz, 2020), or at least five times bigger than NGEU.
3. Once again, the ECB saved the Eurozone, and possibly the EU. ECB intervention at the beginning of the pandemic was based on the experience of the sovereign debt crisis and the ensuing introduction of quantitative easing. The ECB, after initial hesitation, buttressed the euro with the introduction of the €750 billion Pandemic Emergency Purchase Programme (PEPP), which was later increased to €1,850 billion to avoid the dissolution of the Eurozone. PEPP was designed to be flexible, allowing for differentiated intervention based on need. As of 16 April 2021, total (net) purchases under the scheme amounted to €976,5 billion.^6^ The introduction of PEPP bought time to allow the EU Commission and member states to reach an agreement on a joint instrument to pre-empt further crises. Without PEPP, financial bailouts would probably have become unavoidable, as had happened in the Eurozone crisis.

## Economic and political vulnerabilities in the process of EU integration

In this section, we define the key concepts underpinning our empirical analyses. Scholars of EU integration have noted that decisions in the EU have become more politicized since the 1992 Maastricht Treaty (Bremer et al. 2020; Hutter and Grande 2014). Notably, post-functionalism claims that post-Maastricht political actors have had to take more account of their electorates when it comes to matters of EU integration which has created a ‘constraining dissensus’, instead of a ‘permissive consensus’, where elites could take decisions with little, if any, public opposition (Hooghe and Marks 2009). In the context of the pandemic recovery fund, such constraints would have blocked progress towards joint debt liability in the Eurozone as voters in especially Northern countries often fiercely oppose debt mutualisation (Howarth and Schild 2021), while voters in Southern countries tend to be supportive of remaining in the euro, which limits their governments’ bargaining power (Walter et al. 2020). Notwithstanding these polarized positions, past negative experiences prompted EU leaders to engage in a strategy of reconciliation, at least in the run-up to the July 2020 summit, to avoid another existential crisis (Ferrera et al. 2021). Yet, in line with a ‘failing forward’ account of NGEU (Howarth and Quaglia 2021), the establishment of NGEU has taken place in the context of incomplete (and asymmetric) EMU institutional arrangements and their economic and political consequences. This leads Howarth and Quaglia (2021) to conclude that the NGEU was a crisis management response, which did not address the underlying challenges of the asymmetries of EMU. Under this reading, further integration is necessary in order for the EMU—and probably the EU itself—not to fall part. While we agree with such a reading, we also want to highlight how, learning from past failures, EU policy-makers have now acted to prevent the necessity for future—inevitably controversial and expensive—bailouts, which are made likely by the unreformed structure of EMU. In other terms, NGEU addresses the symptoms rather than the cause, but it is undeniable that it does this differently compared to what happened during the *Great Recession*. Also, in our analysis, we highlight the relevance of the political dimension: as Ferrera et al. (2021, p.1347) state: ‘in the post-functionalist era when European integration has become politicised, incumbent leaders are forced to seriously consider the potentially politically undermining implications of distributive politics’.

In what follows, we examine the basis of the distribution of NGEU funds and the impact of the programme on public opinion. Our first concept of interest is *economic vulnerability*, which is the extent to which an economy is structurally weak (as measured by unemployment, public and private debts and public deficits). A worst-case scenario for European policy-makers, both during the Eurozone crisis and under the pandemic, would be that governments with structurally weaker economies would run out of money and fail to mobilize resources at affordable interest rates on international financial markets. While countries outside the Eurozone may adjust the value of their currencies through devaluation, Eurozone countries either face the option of default under these conditions or harsh internal adjustment, with devastating political consequences (Armingeon and Baccaro 2012). We will show that the structure of economic vulnerabilities between EU countries is strikingly similar across the two crises. It is therefore not surprising that during Spring 2020, the pandemic shock posed not only a challenge of economic vulnerability, but also one of *political vulnerability.* This refers to support for or opposition to more European integration in public opinion, which constrains decision-makers. In a situation of ‘constraining dissensus’, politicians are in a precarious position during EU negotiations, since they need to ensure that their parliaments and citizens are appeased, but without undermining the EU project. In other words, in a two-level game setting, governments must balance the agreement of an EU compromise to prevent EU break-up with diverging economic and, relatedly, political interests, which may not allow them to make major concessions at the European level (de la Porte and Jensen 2021).

## Member states vulnerabilities driving NGEU

### Economic vulnerabilities

**Fig. 1:**
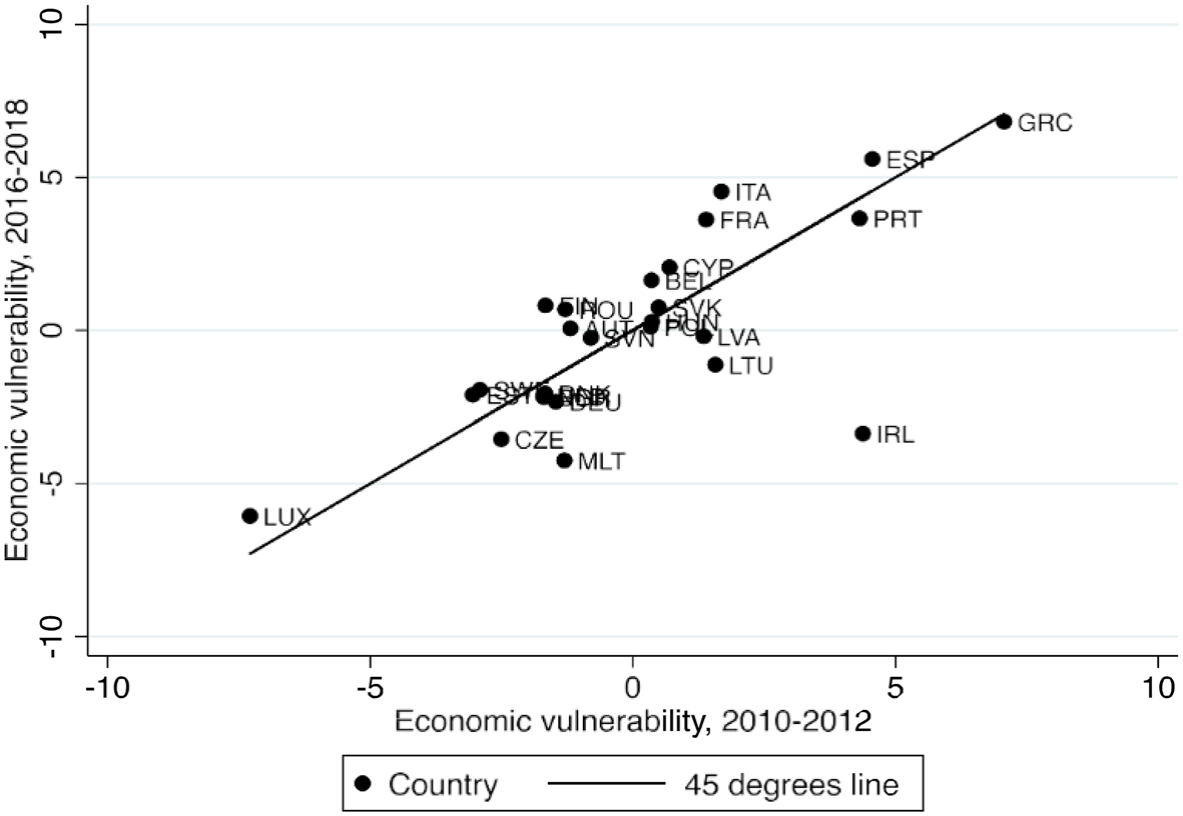
Economic vulnerabilities during the Euro-crisis and immediately before the pandemic

We start from the work of Walter et al. (2020), which combines the level of debts, the size of deficits, the level of unemployment and the private savings of citizens (as % of GDP) into an additive index to measure economic vulnerabilities. For deficits, debts and unemployment with considerable volatility over time, we calculated three-year averages (2010–2012; 2016–2018) to smooth out short-term fluctuations, while for private savings, we used data for 2010 and 2018.^7^ Variables for both time-periods are strongly correlated, with Pearson’s r ranging from 0.91 (debts), over 0.86 (savings) and 0.69 (unemployment) to 0.32 (deficits). On average, the level of debts had increased in 2016–18 as compared to 2010–12 (from 71 to 79% of GDP), while the public balance had improved (from − 4.8 to − 0.4) and savings also grew (from 24 to 28% of GDP) and unemployment fell (from 10 to 8%). Except for when it comes to deficits, all these improvements are far from dramatic in size. On average, EU countries were not much better off before the pandemic as compared to the three worst years of the Eurozone crisis. Likewise, the relative economic vulnerabilities of EU countries did not change dramatically between the Eurozone and the COVID-19 crisis. We calculated the z-scores^8^ for all variables and added up these scores. High scores indicate high vulnerability (Fig. 1).

**Fig. 2:**
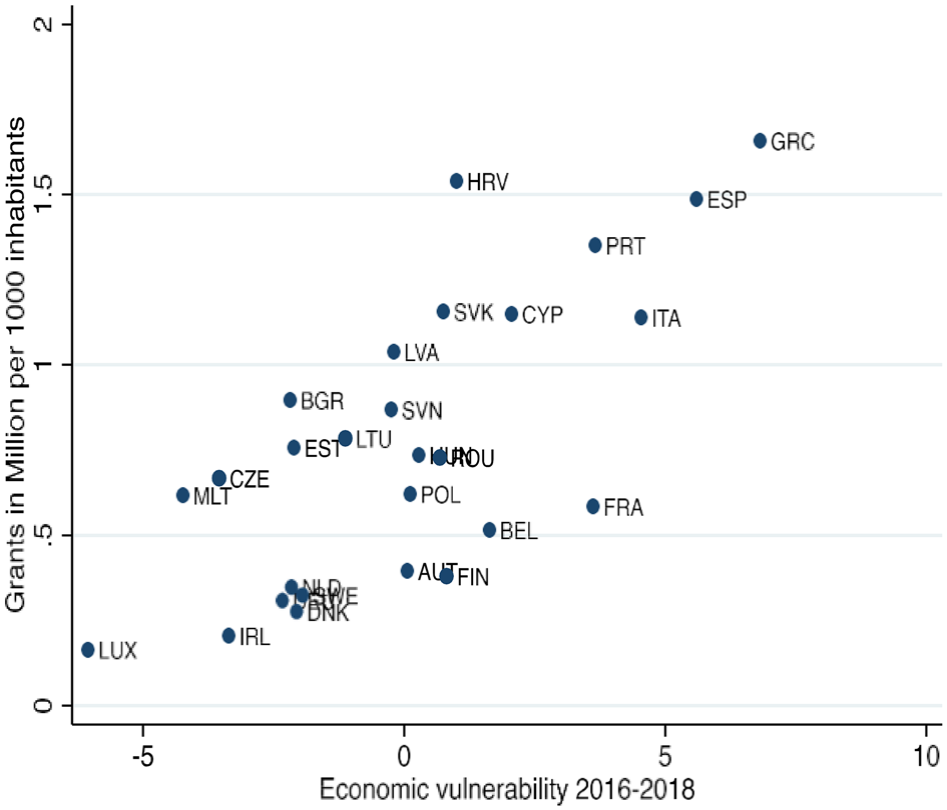
Pre-pandemic economic vulnerability as a correlate of the NGEU grants

As a result, the relative position of countries did not change much between the two periods. EU countries, apart from Ireland, were as vulnerable in 2016–18 as they had been in 2010–12, indicating that the underlying economic problems remained similar. The economies of Portugal, Spain and Greece continued to be weak, while the economies of two big Eurozone states, France and Italy, became more vulnerable. Figure 2 shows that these pre-existing economic vulnerabilities are strongly correlated with generosity of per capita NGEU grants.^9^

To summarize, these data show that economic vulnerability in the EU has not changed much since the Great Recession, and that the more economically vulnerable a member state is, the larger the per capita NGEU grants (first tranche) would be, according to the NGEU allocation key.

### Political vulnerabilities

**Fig. 3:**
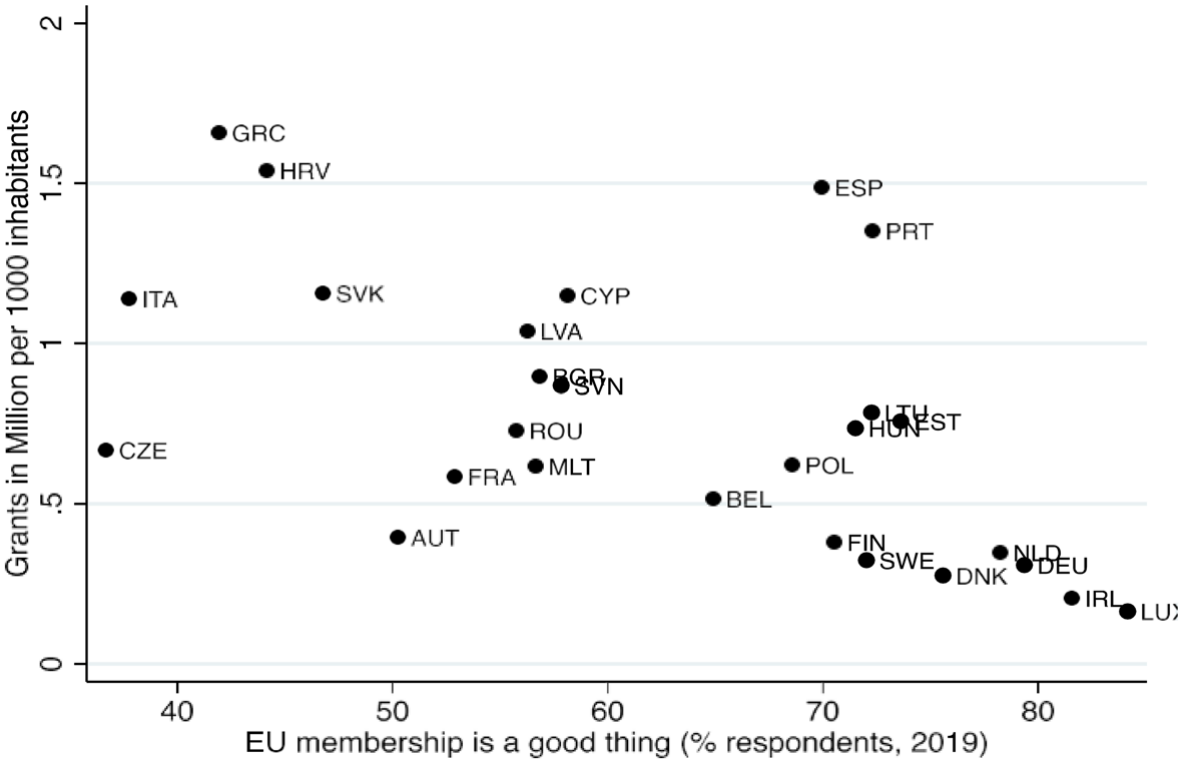
Support of EU and allocation of the NGEU grants

To examine the extent to which distributional decisions were driven by concerns about Euroscepticism, we start with Eurobarometer-data from June/July 2019 (European Commission and European Parliament 2019). We calculate the share of respondents who, prior to the pandemic, thought that their country’s membership of the EU is a good thing. Correlated with receipt of NGEU grants, there is a statistically significant relationship: the larger the share of EU supporters in a country, the lower the earmarked per capita grants from NGEU. This supports the notion that when agreeing on an allocation key, the heads of state and government in the European Council were particularly attentive to the countries with a large share of EU-indifferent or EU-sceptic sentiment (see Fig. 3).

**Fig. 4:**
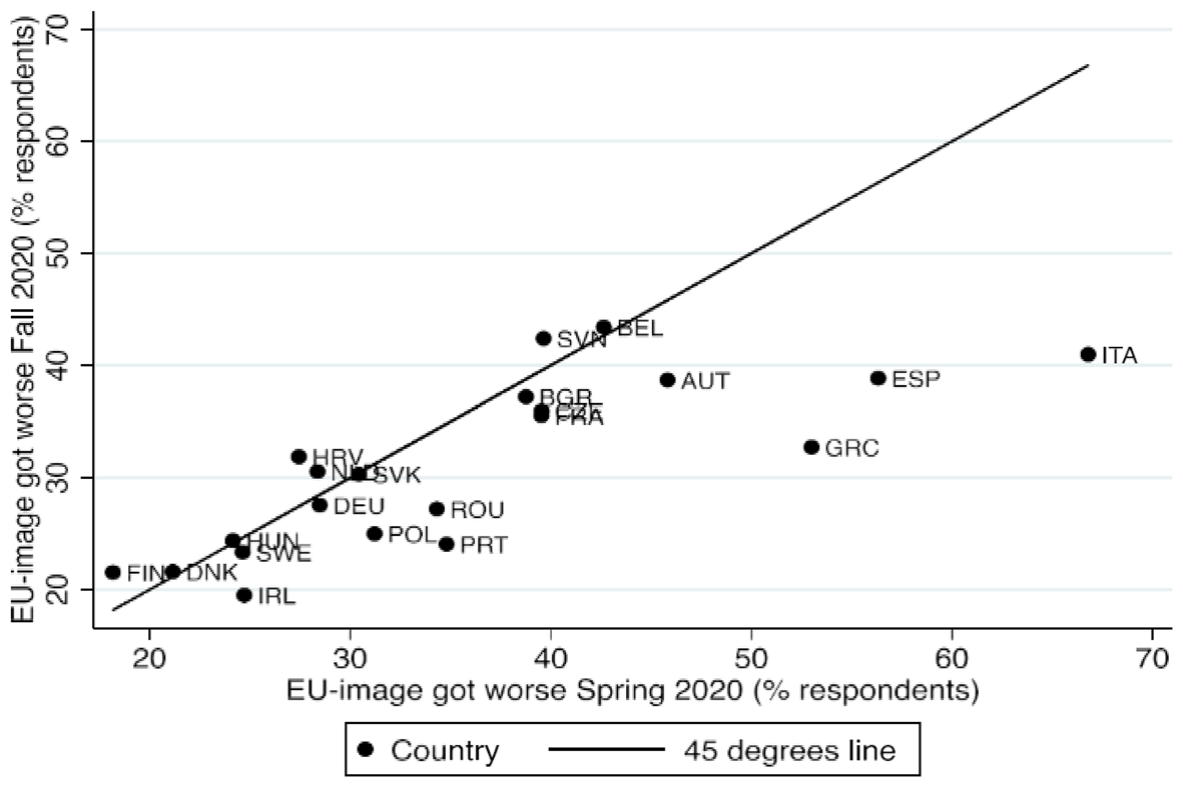
Share of respondents (%) whose image of the EU got worse since the start of the coronavirus pandemic Source: Calculated from European Parliament 2021a, b

The supposition that NGEU was mainly a strategy to soothe Euroscepticism especially in peripheral countries is further supported by surveys during the first months of the pandemic in April/May 2020 and in September/October 2020, not long after the agreement on NGEU in July 2020. Citizens were asked whether their image of the EU had improved, got worse or stayed about the same since the start of the pandemic (see Fig. 4 below).

Figure 4 shows two important facts: (1) At the start of the pandemic, the image of the EU among European citizens deteriorated dramatically for a fifth of the population in Finland and Denmark to half of it in Greece, Spain and Italy. (2) However, after the European Council decision in July 2020, this share receded by a statistically significantly rate in Portugal, Greece, Spain and Italy, which are the countries that have been entitled to the largest NGEU-grants per capita (see also Darvas 2020).

### The relative importance of the severity of the health crisis for economic and political vulnerabilities

**Fig. 5:**
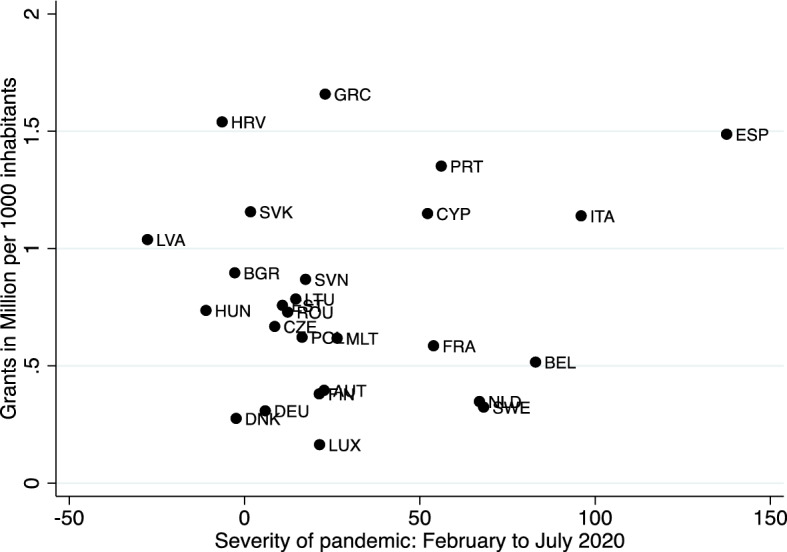
Severity of pandemic and amount of grants

The bivariate correlations presented so far could be spurious, as some economically and politically vulnerable countries—such as Italy and Spain—were hit particularly hard by the first wave of the pandemic. Therefore, we controlled for the severity of the pandemic in the period between the start of the outbreak in Europe in February 2020 and July 2020, when EU policy-makers agreed to establish NGEU. Our control variable is the cumulative monthly excess death rate between February and July 2020^10^ for each country. We deliberately used the data available to policy-makers in Summer 2020, when they took their decisions about NGEU. They could not know then if there would be subsequent waves of the pandemic and they only had access to imperfect data regarding the spread of the virus. However, the excess death rate was known at the time that the negotiations surrounding NGEU took place, which was the most reliable indicator of the severity of the crisis at that time. We find that there is hardly any bivariate correlation between the severity of the pandemic in a member state and the per capita grants earmarked under NGEU (Fig. 5).

Next, we regressed NGEU grants (standardized for population size) on our three independent variables: economic vulnerability, political vulnerability (operationalized through the decline in support for the EU) and severity of the pandemic until July 2020 (Table 1).

**Table 1:** Regression Analysis: NGEU Grants as a function of severity of pandemic, economic and political vulnerability

	M1	M2	M3	M4	M5
Severity of pandemic	0.002			− 0.002	
Economic vulnerability		0.101***		0.095***	0.082***
Political vulnerability			0.017**	0.008	0.010*
_cons	0.743***	0.767***	1.848***	1.307***	1.367***
_*R*_2	0.02	0.52	0.31	0.59	0.60
*N*	26	27	27	26	27

*p < 0.05; **p < 0.01; ***p < 0.001

Comparing the three bivariate models, economic vulnerability has the largest explanatory power (judged by the explained variance) (M2), followed by political vulnerability (M3). If we combine all three variables, the severity of the health crisis for the distribution of NGEU grants is still not significant (M4). Excluding this variable from the models, we once again find a strong effect of economic vulnerability followed by a weaker effect of political vulnerability (M5). Regression diagnostics report no multi-collinearity problems.

This finding supports our claim that NGEU is mainly a reaction to the economic and political vulnerabilities of EU member states prior to the crisis.

## Economic and political vulnerabilities in Italy, Germany and the Netherlands

The previous section has demonstrated how economic and political vulnerabilities have influenced the allocation of NGEU funding. We now turn to examining how such economic and political vulnerabilities unfolded in the run-up to NGEU in three countries: Italy, Germany and the Netherlands. Italy is a first critical case, because rising Euroscepticism in the EU’s third-largest economy would send massive shockwaves throughout the Eurozone and wider EU. Germany has been included as the biggest EU economy whose change in position vis-à-vis financial transfers paved the way for the NGEU package. Finally, the Netherlands, considered the leader of ‘the Frugals’, has an anti-redistributive policy stance due to its domestic economic and political context. Taken together, all case studies show the relevance of economic and political vulnerabilities, and how they constrain and condition leaders, but also how skilful leaders can find room to make deals palatable to their domestic audiences.

### Italy

Italy was the first EU country to introduce a nationwide lockdown in response to the pandemic in early March 2020. Here, we examine the Italian political context surrounding the EU decisions from the onset of the pandemic to the crisis of the government led by Giuseppe Conte (who was in charge at the start of the pandemic) and the formation of a new government led by Mario Draghi in February 2021.

Despite the gigantic economic shock of the pandemic—with industrial production back to the levels of the late 1970s (Confindustria 2020)—Italy’s quest for a relaxation of fiscal rules and the deployment of EU-wide solidarity initially fell on deaf ears. After ECB President Christine Lagarde declared on 12 March 2020 that ‘we are not here to close spreads’, the 10-year Italian paper’s spread with the German equivalent rose from 193 to 263 basis points, the highest single-day hike in history (Financial Times 2020a).

In the following days, the Commission and the ECB acted resolutely. The Commission made structural funds immediately available to fight the pandemic, relaxing state aid rules and proposing to activate the general escape clause in the Stability and Growth Pact. Meanwhile, the ECB introduced the PEPP. On the day PEPP was launched, the spread between Italy’s and Germany 10-year paper fell from 271 to 200 basis points, temporarily easing fears about Italian economic vulnerabilities.

Prior to the European Council meeting on 26 March 2020, Italy strongly advocated for the introduction of Eurobonds, while Conte advocated for the introduction of a common European debt instrument (Financial Times 2020b). In a letter to European Council President Charles Michel, Italy and eight other Eurozone countries called for the issuance of joint EU debt to finance government responses to the pandemic (Financial Times 2020c). When no agreement was reached at the March European Council meeting, Conte threatened not to support the concluding statement, recoiling only when Michel adjourned the summit and entrusted the Ecofin Council with the task of making proposals to address the crisis (Politico 2020a).

The Italian prime minister’s position must be understood against the backdrop of the unwavering opposition of the largest party in his supporting coalition, the populist Five Star Movement (M5S), to any possibility of using the European Stability Mechanism (ESM) as response to the pandemic, even if only conditional on earmarking funds for health expenditure. While the other major coalition partner in the government, the centre-left Democratic Party (PD), was in favour of such a ‘pandemic’ ESM, M5S made clear that this would be a non-starter for them, and would possibly bring about the end of the government. Strategically, M5S had to beware the right-wing populist League party, which had been gaining support in the opinion polls before the pandemic, while support for M5S had fallen. The position of the League’s leader, Matteo Salvini, was forthright: ‘I do not want to hear the word ESM again’, calling conditionality ‘a criminal idea’ (Il Fatto Quotidiano 2020), pointing to the loss of sovereignty attached to asking for ESM support.^11^

Conte also had to face divided public opinion: in April 2020, Italian respondents were evenly split on the issue of accepting financial aid from the EU, even if this would come with conditions, amid strong partisan cleavages. Two thirds of voters of right-wing parties (including the League) stood against accepting conditional aid, as opposed to 26% of PD voters, while M5S voters were evenly split (DISPOC/LAPS and IAI 2020a).

While the Italian government backed away from any possibility of tapping into even a reformed ESM due to domestic political constraints, it increased the 2020 budget deficit to more than 10% of GDP in order to fund a compensation package for workers and companies, with an estimated impact on the debt/GDP ratio of 155.7% in 2020, as compared to a pre-pandemic projected deficit of 1.8% of GDP for 2020 (Servizio studi Senato e Camera 2020) and a debt/GDP ratio of 134.8% in 2019 (Eurostat 2020).

After the Eurobond proposal had been removed from the negotiation table (Schelkle forthcoming), the Italian government supported the proposal by European Commissioners Paolo Gentiloni (Italy, Economic affairs) and Thierry Breton (France, Internal market) to introduce a €1,500 billion “purpose-built European fund that could issue long-term bonds”. This was the embryo of NGEU, although its economic magnitude was trimmed down.

When the battle surrounding NGEU started to revolve around the issue of grants as opposed to loans, Italy sided with French President Macron’s demand for ‘real budgetary transfers’, that is, grants instead of loans. When the European Commission, following the Franco-German initiative, proposed a balance between grants and loans in May 2020, amounting to a total of 750 billion euros, Conte adjusted his public discourse and called it ‘an excellent signal from Brussels’ (ANSA 2020), stating that although it was not the proposal Italy had envisioned, it was adequate and could contribute ‘to distract morbid attention surrounding the ESM’ (Reuters 2020).

The outcome of the negotiations on NGEU has bolstered support for the EU among Italians. When polled in October–November 2020, the share of respondents that would vote to leave the EU had fallen from 48% in April 2020 to 37%, and those that would vote to remain had increased from 44 to 56%. The highest support comes from the PD (92% supporting remaining in the EU) and other left-of-centre party voters (95%), with the lowest support being among League voters (31%), with M5S voters falling in between (56%). Also, the feeling of Italy being treated unfairly in budgetary matters fell from 69% in April 2020 to 49% after the introduction of NGEU (DISPOC/LAPS and IAI 2020b).

After July 2020, tensions within the governing coalition over the direction of governmental action increased, as did the coalition partners’ dissatisfaction with Conte’s strategy to avoid any potentially conflictual decisions. From being seen as a personal success of the prime minister, the RRF became his government’s bane. The drafting of the plan was kept in the hands of the prime minister and not circulated until December 2020 (Politico 2021). This first draft was deemed unsatisfactory by the coalition partners, especially former PD prime minister Matteo Renzi, who was also frustrated with the ESM not being used. When Renzi, who had left the PD in September 2019 to found a new social liberal party, withdrew his party’s support, the government fell and was replaced by a grand coalition including all parties, except the far-right party Brothers of Italy. The new government was headed by a technocrat, the former ECB president Mario Draghi, who installed non-partisan civil servants and managers in some key government positions. The League joined the government, toning down the party’s populist rhetoric to the benefit of the interests of many in the historic heartlands of its forerunner party, the Northern League, in the productive North, that saw the money of the RRF as the last chance of staying within Europe’s core, and the drawing up of the plan as a window of opportunity for introducing liberalising reforms. Alongside serving strategic purposes—demonstrating reliability and responsibility to other European governments—the League’s “EU-turn” testifies to Italy’s enduring economic vulnerabilities, and—in parallel with the events of late 2011 in the Eurozone crisis (Sacchi 2015)—to their constraining power over domestic political struggles.

### Germany

During the Eurozone crisis, the German government was strictly against any common debt issuance and the country’s leaders were apportioned much blame for the harsh austerity stance of the ‘Troika’. Particularly Chancellor Angela Merkel was much maligned (Crespy 2020) for claims that Greece and other Southern European countries had indulged in fiscal profligacy and had ‘not done their homework’, whereas Germany had removed rigidities in its labour market and had reformed its unemployment benefit system with the sweeping Hartz reforms in the early 2000s (Armingeon and Baccaro 2012). The welfare retrenching effects of the Hartz reforms made the prospect of ‘paying other countries’ debt’ with German money unpalatable domestically and any impression to be the ‘paymaster’ for the Eurozone was a political vulnerability that could be exploited by populist opponents in the domestic context. In fact, the measures that were eventually taken to rescue the Eurozone led to the formation of the right-wing populist challenger party Alternative für Deutschland (AfD) in 2013.

During and after the Eurozone crisis, it became undeniable that the export-oriented and highly competitive German economy continued to benefit enormously from the opportunities offered by EMU membership. The country was attractive for investors and thus profited from low interest rates on their sovereign bonds (Armingeon et al. 2016). However, the contradictions within the EU’s growth model and institutional architecture that has worked to Germany’s advantage so far are not in the interest of the country in the long-term and forced it to change its position when the COVID-19 pandemic hit (Celi et al. 2020). Initially, the German preference was to rely on the ESM as a vehicle for supporting EU economies particularly hit by the pandemic, but with the usual ESM conditionality replaced by a softer health-related requirement as a gesture of solidarity (Politico 2020a). Important in this respect is that moral hazard allegations that dominated the political discourse during the Eurozone crisis found it harder to surface during the pandemic, given the different characters of this crisis (Buti 2020).

Over the initial months of the pandemic, Germany changed its stance from categorically excluding joint debt liability to supporting, as of May 2020 (together with France), a common EU recovery fund (Bundesregierung 2020). This shift of position has to be understood in light of the *economic vulnerabilities* emanating from the threat of further disruption in EMU and the possible collapse of the Eurozone. Merkel’s surprising shift partly stemmed ‘from a recognition of the steep risk to the EU posed by glaring differences in how countries were positioned to respond to the crisis’ (Politico 2020b). Merkel was cited in May 2020 as having said that, ‘It is essential for Germany, as an export nation, that its EU partners also do well’ (Euractiv 2020a). Her Christian Democratic party budget spokesperson confirmed this pragmatic view of the crisis measures when he exclaimed in Parliament that Germany will benefit most, ‘even if we pay four times as much as we get back’ (Metz 2021a).

Some important context to this change of position is that in the aftermath of the Great Recession and Eurozone crisis, when Southern Europe was weak, Germany’s export industry mainly relied on the markets of the UK, the US, and particularly China. However, international trade and global value chains have been disrupted since 2016 due to the economic uncertainties relating to factors including Brexit, President Trump’s protectionist policies, and changes in Chinese economic policy. Germany’s export-led growth has therefore become much more reliant on its export markets in Southern Europe as well as on manufacturers in the East and South of Europe to sustain its own production and to secure the EU’s long-term sustainability (Buti 2020; Celi et al. 2020).

The economic vulnerability of Southern Europe thus became a threat to Germany’s economic model as much as the ensuing Euroscepticism in the South became a political risk for European integration, the latter a longstanding hallmark of German policy: ‘For us in Germany, the commitment to a united Europe is part and parcel of our reason of state’, (Merkel, quoted in Ferrera et al. (2021), p.1346). The Merkel-Macron plan of May 2020 was therefore not a case of pure altruism but rather an example of ‘self-interested solidarity’. As Celi et al (2020, p. 420) argue, ‘[b]ehind the good intentions, there are the concrete interests of both France and Germany for the survival of the EMU: they look with growing concern at the rise of Euroscepticism in the SP [Southern Periphery]’.

Political vulnerabilities are still relevant even though at the time, the Eurosceptic AfD was posing only a mild threat as it was hampered by infighting (NPR 2020) and threatened with investigation by the domestic intelligence agency at the peak of the pandemic (Financial Times 2021). German public opinion has also been relatively supportive of the joint EU effort. According to a Eurobarometer poll in October 2020, of those who have heard about the measures, 7% are very satisfied, 47% fairly satisfied and only 9% not at all satisfied with them (European Parliament 2020). A survey experiment by Baccaro et al (2021) found that a majority of German voters, once they received information about the potential consequences of a break-up of the Eurozone due to ‘Italexit’ (i.e. Italy’s departure from the currency union), favour debt mutualisation. Based on these findings, the authors argue that public opinion needs not be an inevitable binding constraint for more financial risk-sharing across the Eurozone.

However, a political constraint is exerted by the German Constitutional Court. Even if public opinion and a parliamentary majority are in favour of new measures that would pre-empt another ex-post bailout, it only needs a few dissenters to call on the Court to potentially intervene in a way that would stop any EU-level agreement in its tracks, as demonstrated in Spring 2021 (Bundesverfassungsgericht (2021); see also Bulmer (2021), this issue). Indeed, the coalition of Christian Democrats (CDU) and Social Democrats (SPD) is split over the issue of common debts: while the SPD would not object to a permanent fiscal union, the CDU only views the new instruments to be permissible as temporary crisis measures and is at pains to emphasise their exceptional character (Metz 2021a, b). In addition, to ward off any populist challenges while selling the idea of NGEU at home, it is worth noting that German liability for common debts is limited and Germany was the only non-frugal country to secure a significant rebate in its contribution to the EU budget as part of the wider NGEU negotiations (de la Porte and Jensen 2021).

### The Netherlands

The change of Germany’s position meant that the remaining Northern countries advocating fiscal responsibility in the EU had to forge a strong alliance. The Netherlands, spearheaded by the Liberal Mark Rutte, became the lead of the ‘Frugals’, a group of export-oriented, small, rich and well-coordinated countries with low levels of public debt and good macro-economic health, also including Denmark, Sweden and Austria that maintained an antagonistic position towards debt mutualisation in the build-up to the NGEU compromise (Ferrera et al. 2021). The consistent position of the Netherlands was that irrespective of the type of instrument agreed at EU level, there would have to be strong conditionality. Rutte’s long-standing EU policy has been characterised by Otjes (2021:70) as ‘a brake on any step that would transform the EU into a transfer union between economically stronger Northern European countries and economically weaker Southern countries’.

When a ‘pandemic’ credit line within the ESM started to be discussed, Rutte argued that a general reference to respect for the EU fiscal rules in the ESM would not be sufficiently stringent and that the ESM should be considered only as a lender of last resort (Euractiv 2020b). In the end, the ESM Pandemic Crisis Support did not involve any country-specific conditionality related to the introduction of structural reforms. This was acceptable to Rutte, because the instrument was a loan and therefore did not cause major tension in his own national parliament.

With regard to NGEU, the red line in the frugals’ position related to common debt issuance and grants, since all four feared the prospect of a fiscal union (Frugals non-paper 2020). Furthermore, they were vocal about specifying conditionality related to economic reforms in the final negotiations of the European Council. The Dutch government, notably Dutch Finance minister Wopke Hoekstra from Rutte’s coalition partner CDA (Christian Democratic Appeal), proposed a model similar to Memorandums of Understanding in the sovereign debt crisis, whereby access to payments would be conditional on successful progress towards specified targets and reforms (Government of the Netherlands 2020). The Netherlands was also concerned about the capability of the Southern European countries to use the money effectively. In the end, the demands from the Netherlands were only met with regard to process, since the Council conclusions specify that countries would have to meet milestones and targets. However, as plans are to be linked to CSRs under the European Semester, economic reforms will be a requirement, especially for countries with economic vulnerabilities. The Dutch Prime Minister also wanted the inclusion of a clause specifying that one member state could veto the disbursement of funding; however, these demands were softened in the Council compromise. In exchange for accepting the grant instrument, the Netherlands negotiated a considerable rebate in its contribution to the EU budget.

The strong line on conditionality was a continuation of the EU policy of Rutte’s own liberal-conservative party, VVD. The CDA has a similar centrist position, supporting European integration, while taking a tough line on the responsibilities of Southern European ‘debtor countries’ (Witteven 2017). Rutte’s insistence on the attachment of conditionality provisions in the run-up to the Council negotiations was also crucial for disarming the populist Eurosceptic Freedom party (PVV) of Geert Wilders (Otjes 2021). Some scholars argue that a strong stance on southern Europe, which emerged during the sovereign debt crisis, has spread from the PVV to the more centrist parties in Dutch politics (Oudenampsen 2018). Indeed, after the NGEU was agreed, Rutte faced criticism from the PVV that the grant instrument had been accepted, while opposition parties criticised Rutte for not showing more solidarity (Politiken 2021).

A survey from October 2020 revealed that the majority of citizens in frugal states view the NGEU deal as largely positive, although there are concerns regarding potential corruption in the use of funds and regarding the need for oversight (Dennison and Zerka 2020).

## Discussion and conclusion

NGEU has been hailed by some as a qualitative change in the EU, a historic achievement (Jones 2021) and a case of transformative policy learning (Ladi and Tsarouhas 2020). Others take a more negative view and argue that NGEU is a case of ‘failing forward’ as the EU has failed to solve existing asymmetries and structural problems left over from the previous Eurozone crisis and have possibly sowed the seeds of future crises with the achieved compromise (Howarth and Quaglia 2021).

In this paper, we have shown that NGEU can be seen as an ex-ante intervention to avoid another humiliating and conflict-ridden bailout as pursued in the previous major crisis of the EU in 2010–2015. It echoes prior imbalances that were created and amplified by the Great Recession, due to the establishment of a common currency without permanent fiscal redistribution to structurally weaker economies. Those countries, which would be most vulnerable to more adjustments by austerity after the economic crisis induced by the pandemic, receive most resources — as we argue, precisely to avoid or mitigate such costly adjustments. It can also be seen as a reaction to political vulnerabilities that inevitably follow from economic vulnerabilities and tensions. Those countries with strong anti-EU sentiment have been entitled to particularly large NGEU grants per capita. In contrast, NGEU grants are not correlated with the severity of the health crisis in the first half of 2020.

Conflicts among self-interested actors shaped NGEU. The Commission used the window of opportunity to push forward its plans for green and digital innovation. As shown in our qualitative case studies, the Netherlands, as the leader of the frugal member states, wanted to avoid the distribution of grants and insisted on conditionality to appease its domestic audience. Germany wanted to avoid a populist backlash in Southern Europe and, importantly, wanted to protect its own economic interests in a functioning Eurozone and to safeguard key export markets for its products. Italy resisted the lure of ESM loans, even with less conditionality than during the sovereign debt crisis, due to their bad reputation among the national electorate and their potential stigma, and instead fought for grants without conditionality to avoid providing ammunition to the populist right.

While this all resonates with a failing forward argument against the backdrop of post-functionalist constraining dissensus, we also recognize that learning took place, albeit in a limited sense: the overall EU fiscal architecture remains unchanged, but the EU introduced NGEU as an innovation to avoid future bailouts. At the same time, while highlighting political—alongside economic—vulnerabilities and constraints, our case studies also show that political leaders retain room for manoeuvre in steering consensus (Ferrera et al. 2021). Although we cannot deny the innovative potential of NGEU (Rhodes 2021), the evidence put forward in this paper shows that for the time being at least, NGEU is a politically constrained solution to address the mess created over the previous decade, and as such it is—at best—a Janus solution: promising a fresh start, but haunted by the past.

## Footnotes

1 The Commission’s plans for obtaining investment commitment for the green and digital agendas date back to the. Juncker Commission. In this regard, the pandemic offered a window of opportunity to foster what is presented as a new European growth model.

2 The NGEU comprises seven distinct sub-programmes (RRF, ReactEU, Just Transition Fund, EAFRD, rescEU, Horizon Europe, InvestEU). RRF amounts to €672.5 billion (€360 billion in loans and €312.4 billion in grants) out of the overall package of €750 billion.

3 Throughout this paper, we analyse the NGEU as an EU-wide programme, which is however particularly relevant when seen through the lenses of economic integration and its strongest form of institutionalization, the Economic and Monetary Union (EMU). We posit that if a country were to exit the Eurozone, this would likely lead to a break-up of the EMU and, possibly, the EU as a whole.

4 The first hint of NGEU came from the letter of Commissioners Gentiloni and Breton to several European newspapers in early April 2020. They advocated a stimulus size comparable to that of Germany, around 10% of GDP. This would have amounted in their account to €1500–1600 billion, ‘to be injected directly into the economy’ (The Irish Times, 6/4/2020). Two Liberal MEPs, Garicano and Verhofstadt, had also proposed the introduction of a “European Reconstruction Fund” (Schelkle forthcoming).

5 The Recovery and Resilience plans of member states have been submitted on 30 April, showing that all plans thus far apply fully for grants, but application for loans is mixed: https://ec.europa.eu/info/business-economy-euro/recovery-coronavirus/recovery-and-resilience-facility_en.

6 https://www.ecb.europa.eu/mopo/implement/pepp/html/index.en.html, accessed April 25, 2021.

7 Sources are Armingeon et al. (2020), Comparative Political Data Set 1960–2018, available at http://www.cpds-data.org/ and the World Bank’s World Development Indicators, available at https://databank.worldbank.org/source/world-development-indicators.

8 z-standardization transforms a distribution to a mean of zero and a standard deviation of one, so that distributions with very different original figures can be easily compared.

9 Grants as of July 2020, standardized by the size of population. https://ec.europa.eu/info/sites/default/files/about_the_european_commission/eu_budget/recovery_and_resilience_facility_.pdf, accessed 10 March 2021.

10 This figure expresses excess mortality as a percentage increase of the average number of deaths in the same month of the baseline period 2016–2019. https://ec.europa.eu/eurostat/statistics-explained/index.php?title=Excess_mortality_-_statistics).

11 Despite the only conditionality of the ESM pandemic credit line being the earmarking of funds for health-related expenditures, the strong association of the ESM with austerity programmes during the Eurozone crisis made this option very divisive in the Italian public debate. Moreover, accessing ESM—which also enjoys preferred creditor status—might be seen as an advance declaration of potential market access problems, thereby entailing stigma.
